# Evaluation of renal function in a patient with terminal rectal cancer who developed morphine hydrochloride–induced respiratory depression

**DOI:** 10.20407/fmj.2025-021

**Published:** 2026-05-14

**Authors:** Yoshihiro Uekuzu, Masanobu Usui, Akihiko Futamura, Takahiko Inagaki

**Affiliations:** 1 Department of Pharmacy, Fujita Health University Okazaki Medical Center, Okazaki, Aichi, Japan; 2 Department of Surgery and Palliative Medicine, Fujita Health University, School of Medicine, Toyoake, Aichi, Japan; 3 Faculty of Pharmaceutical Science, Suzuka University of Medical Science, Suzuka, Mie, Japan

**Keywords:** Terminal cancer patient, Sarcopenia, Serum cystatin C, eGFR, Morphine

## Abstract

A challenge in patients with terminal cancer is that serum creatinine–based estimates of renal function often deviate from true renal function because cachexia-related loss of muscle mass leads to abnormally low serum creatinine levels. Serum cystatin C is a useful marker of renal function in patients with decreased muscle mass; however, there are limited data on its use in patients with terminal cancer. In this study, we present a detailed assessment of renal function, incorporating muscle mass measurement by bioelectrical impedance analysis, in a patient with terminal rectal cancer who developed respiratory depression while receiving a stable dose of morphine hydrochloride injections.

## Introduction

To ensure appropriate drug therapy in patients with cancer, it is essential to assess cancer stage, expected survival duration, general condition, and organ function. These factors help determine the optimal drug and method of administration. Opioids are highly effective for alleviating certain symptoms in patients with cancer; however, they carry a risk of respiratory depression, a serious adverse effect associated with overdose that necessitates careful monitoring.^1^ In particular, morphine is effective for alleviating dyspnea and pain in patients with cancer; however, in those with renal dysfunction, cautious use is required because its active metabolites such as morphine-6-glucuronide (M6G) and morphine-3-glucuronide (M3G) accumulate, necessitating dose reduction and careful monitoring.^2,3^ In general, equations that estimate glomerular filtration rate based on serum creatinine are recommended for the evaluation of renal function in clinical practice. However, this method is limited by the fact that serum creatinine levels are influenced by changes in muscle mass. Recently, cachexia, which occurs in patients with terminal cancer, has been identified as a pathological condition marked by a substantial loss of muscle mass.^4^ Consequently, routine use of serum creatinine–based estimated glomerular filtration rate (eGFR) equations in these patients may overestimate renal function. In contrast, eGFR equations based on serum cystatin C may provide a more accurate assessment of renal function in patients with reduced muscle mass, as cystatin C levels are less affected by muscle mass changes.^5^ Therefore, to assess renal function accurately in patients with terminal cancer, it is important to consider individual factors, such as muscle mass, and to select the most appropriate eGFR equation for each case.

In this report, we present a detailed assessment of renal function, incorporating muscle mass measurement using bioelectrical impedance analysis (BIA), in a patient with terminal rectal cancer who developed respiratory depression while receiving a stable dose of morphine hydrochloride.

## Methods

### Subject

Patient: A man in his 70s

Illness: Rectal cancer

Metastatic sites: Liver, lungs, and lymph nodes

Past medical history: Cerebral hemorrhage

History of the present illness: In February X–2, the patient underwent an abdominoperineal rectal resection for rectal cancer. In June of that year, the patient began chemotherapy for liver and anterior sacral lymph node metastases resulting from recurrent rectal cancer. In January X–1, chemotherapy was suspended because of a cerebral hemorrhage and was resumed in December of the same year. Subsequently, chemotherapy was discontinued because of disease progression and worsening of the patient’s general condition. In January X, the patient was admitted to our hospital for palliative care.

Sarcopenia assessment: The patient was 170 cm tall, weighed 48.7 kg, and had a body mass index (BMI) of 16.9 kg/m^2^. His skeletal muscle mass index, measured by BIA (InBody S10, InBody Japan Inc.), was 5.4 kg/m^2^, below the cutoff value of 7.0 kg/m^2^, leading to a diagnosis of sarcopenia.^6^

Clinical course: At the time of hospitalization, the patient was fasting and receiving parenteral nutrition. He spent most of the day in bed due to numbness in both lower limbs, a residual effect of the cerebral hemorrhage. Considering his advanced cancer and a palliative performance scale score of 20, his life expectancy was estimated at approximately one month, leading to a diagnosis of terminal cancer.^7,8^ Fentanyl patches (2 mg/day) were administered for visceral abdominal pain but did not achieve adequate pain relief. Accordingly, the opioid regimen was switched to morphine hydrochloride injections (20 mg/day) on the day of hospitalization. Thereafter, the patient’s cancer pain ranged from 1 to 5 on the numerical rating scale and was relatively well controlled; thus, treatment with morphine hydrochloride injections was continued at the same dose. On day 21, the patient exhibited a reduced respiratory rate (<8 breaths/min), coma, and cyanosis. Respiratory depression due to morphine overdose was suspected, and the patient’s condition was considered life-threatening. A naloxone hydrochloride injection was administered, resulting in rapid improvement of the respiratory depression.^9^ The exact urine output at the onset of respiratory depression could not be determined because a bladder catheter was not in place; however, there were no signs of fluid retention, such as edema, pleural effusion, or ascites. Between hospitalization and the onset of respiratory depression, the patient received only morphine hydrochloride injections, total parenteral nutrition, fat emulsion, famotidine injections, and prochlorperazine injections. Following the respiratory depression episode, the opioid regimen was switched to fentanyl injections (0.4 mg/day), with no subsequent withdrawal symptoms. The patient died from progression of the primary disease on day 34.

### Renal function assessment

We assessed renal function at the time of and after initiation of morphine hydrochloride injections, including measurements taken before and at the onset of respiratory depression. Renal function was evaluated using serum creatinine, measured by an enzymatic method (LABOSPECT 008 α, Hitachi High-Tech Corporation) with L-type Wako CRE-M reagents (Fujifilm Wako Pure Chemicals Corporation), with a reference range of 0.65–1.07 mg/dL. We also measured serum cystatin C using residual samples collected after the onset of respiratory depression. Measurements were performed using a latex immunoturbidimetric assay (LABOSPECT 008 α, Hitachi High-Tech Corporation) with Norudia Cystatin C reagents (Sekisui Medical Co., Ltd.), with a reference range of 0.58–0.98 mg/L. These data were used to calculate eGFR based on serum creatinine and cystatin C and to examine their relationship with the development of respiratory depression over time. We used the following four equations to calculate eGFR:

Equation for eGFR based on serum creatinine (eGFR_Scr_) in Japanese patients^10^


$$\begin{array}{c} {\mathrm{eGFR}}_{\mathrm{Scr}}\;\left(\mathrm{mL}/\min/1.73\;m^{2}\right)\\ \;=194\times \mathrm{serum}\;\mathrm{creatinine}\;{\left(\mathrm{mg}/\mathrm{dL}\right)}^{-1.094}\times \mathrm{age}\;{\left(\mathrm{years}\right)}^{-0.287}\\ \;\times 0.739\;\left(\mathrm{for}\;\mathrm{females}\right) \end{array}$$

Equation for eGFR based on serum cystatin C (eGFR_Scys_) in Japanese patients^11^


$$\begin{array}{c} {\mathrm{eGFR}}_{\mathrm{Scys}}\;\left(\mathrm{mL}/\min/1.73\;m^{2}\right)\\ \;=\{104\times \mathrm{serum}\;\mathrm{cystatin}\;C\;{\left(\mathrm{mg}/L\right)}^{-1.019}\times 0.996^{\mathrm{age}\;\left(\mathrm{years}\right)}\\ \;\times 0.929\;\left(\mathrm{for}\;\mathrm{females}\right)\}\;-8 \end{array}$$

Equation for eGFR based on the combination of serum creatinine and serum cystatin C (eGFR_Scr,_ _Scys_) in Japanese patients^11^


$$\begin{array}{c} {\mathrm{eGFR}}_{\mathrm{Scr},\mathrm{Scys}}\;\left(\mathrm{mL}/\min/1.73\;m^{2}\right)\\ \;=\frac{{\mathrm{eGFR}}_{\mathrm{Scr}}\;\left(\mathrm{mL}/\min/1.73\;m^{2}\right)+{\mathrm{eGFR}}_{\mathrm{Scys}}\;\left(\mathrm{mL}/\min/1.73\;m^{2}\right)}{2} \end{array}$$

Corrected eGFR from the Cockcroft–Gault equation (eGFR_CG_)^10,12,13^


$$\begin{array}{c} {\mathrm{eGFR}}_{\mathrm{CG}}\;\left(\mathrm{mL}/\min/1.73\;m^{2}\right)\\ \;=\frac{\left\{140-\mathrm{age}\;\left(\mathrm{years}\right)\right\}\times \mathrm{weight}\;\left(\mathrm{kg}\right)}{72\times \mathrm{serum}\;\mathrm{creatinine}\;\left(\mathrm{mg}/\mathrm{dL}\right)}\times \frac{1.73}{\mathrm{body}\;\mathrm{surface}\;\mathrm{area}}\;\left(m^{2}\right)\\ \;\times 0.789\;\left(\mathrm{correction}\;\mathrm{factor}\right)\times 0.85\;\left(\mathrm{for}\;\mathrm{females}\right) \end{array}$$

### Ethical considerations

This study was conducted in accordance with a protocol approved by the Ethical Review Board of Fujita Health University.

## Results

**Figure 1:**
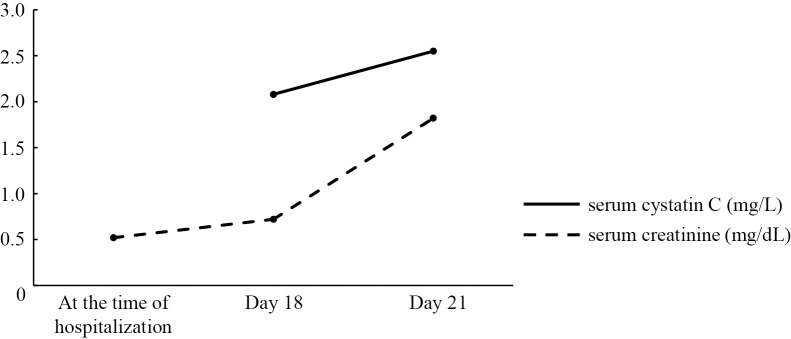
Changes over time in renal function (serum creatinine and serum cystatin C) and the occurrence of respiratory depression At the time of hospitalization: At the initiation of morphine hydrochloride injection Day 18: Three days before the onset of respiratory depression Day 21: At the onset of respiratory depression

**Figure 2:**
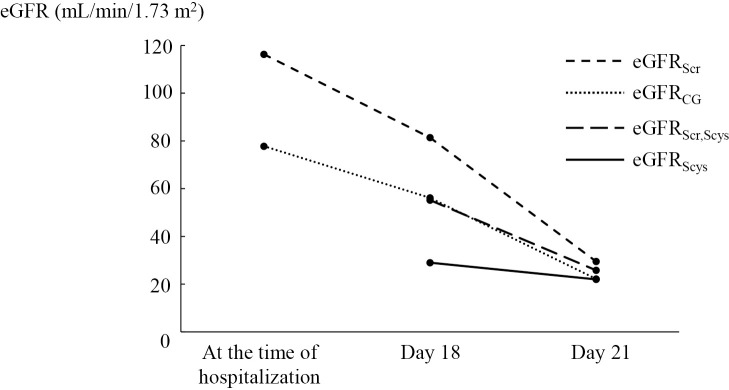
Changes over time in renal function (eGFR) and the occurrence of respiratory depression At the time of hospitalization: At the initiation of morphine hydrochloride injection Day 18: Three days before the onset of respiratory depression Day 21: At the onset of respiratory depression eGFR: Estimated glomerular filtration rate (mL/min/1.73 m^2^) eGFR_Scr_: Equation for eGFR based on serum creatinine in Japanese patients eGFR_CG_: Corrected eGFR values using the Cockcroft–Gault equation eGFR_Scr, Scys_: Combined equation for Japanese patients using serum creatinine and serum cystatin C eGFR_Scys_: eGFR equation for Japanese patients based on serum cystatin C

In this study, serum cystatin C was measured following the onset of respiratory depression. However, serum cystatin C could not be measured at the initiation of morphine hydrochloride injection, as no residual sample was available. Figure 1 presents the measured serum creatinine and serum cystatin C levels. Serum creatinine levels (mg/dL) at the initiation of morphine hydrochloride injection and before and at the onset of respiratory depression were 0.52, 0.72, and 1.82, respectively. At the corresponding time points, serum cystatin C levels (mg/L) were unmeasurable, 2.08, and 2.55. Figure 2 shows eGFR values (mL/min/1.73 m^2^) calculated using the different equations. At the initiation of morphine hydrochloride injection and before and at the onset of respiratory depression, eGFR values calculated using the serum creatinine–based equation for Japanese patients (eGFR_Scr_) were 116.3, 81.4, and 29.5 mL/min/1.73 m^2^, respectively. In contrast, eGFR values calculated using the serum cystatin C–based equation for Japanese patients (eGFR_Scys_) were unmeasurable at initiation and were 29.0 and 22.0 mL/min/1.73 m^2^ before and at the onset of respiratory depression, respectively. eGFR_Scys_ indicated renal dysfunction at least 3 days before the onset of respiratory depression, whereas eGFR_Scr_ overestimated renal function. Using the equation for eGFR based on the combination of serum creatinine and serum cystatin C in Japanese patients (eGFR_Scr,Scys_), values were unmeasurable at initiation and were 55.2 and 25.8 mL/min/1.73 m^2^ before and at the onset of respiratory depression, respectively. Corrected eGFR values calculated using the Cockcroft–Gault equation (eGFR_CG_) were 77.8, 56.2, and 22.2 mL/min/1.73 m^2^ at the respective time points. Overall, eGFR equations incorporating serum creatinine or the Cockcroft–Gault equation tended to overestimate renal function.

## Discussion

This study presents a detailed evaluation of renal function, incorporating muscle mass measurement using BIA, in a patient with terminal rectal cancer who developed respiratory depression while receiving a stable dose of morphine hydrochloride injections. The findings suggest that serum cystatin C–based assessment of renal function may be useful for the appropriate use of morphine in patients with terminal cancer.

The most important finding of this study is that eGFR equations based on serum creatinine or the Cockcroft–Gault equation overestimate renal function in patients with terminal cancer and sarcopenia. In general clinical practice, renal function can be accurately estimated using serum creatinine alone or in combination with serum cystatin C measured in blood tests.^10,11^ However, eGFR_Scys_ is considered useful for accurately assessing renal function in patients with terminal cancer who have cachexia and marked muscle mass loss, as it does not rely on an equation that incorporates serum creatinine.

A second important finding is that 3 days before the onset of respiratory depression, eGFR_Scys_ was 29.0 mL/min/1.73 m^2^, indicating renal dysfunction, whereas eGFR_Scr_ was 81.4 mL/min/1.73 m^2^, markedly overestimating renal function. The discrepancy between eGFR_Scys_ and eGFR_Scr_ was 52.4 mL/min/1.73 m^2^, a difference likely to have a substantial effect on clinical decision-making regarding opioid selection and dosing. The guidelines recommend avoiding morphine in patients with renal dysfunction whenever possible and, when its use is unavoidable, administering it at a reduced dose or with an extended dosing interval.^2^ This patient developed respiratory depression while receiving a stable dose of morphine hydrochloride. If serum cystatin C had been used to detect renal dysfunction at an earlier stage, proactive measures such as reducing the morphine dose or switching to an alternative opioid could have been implemented. Furthermore, morphine clearance has been reported to decline during the terminal phase, highlighting the importance of early detection of renal dysfunction in drug therapy.^14^

Notably, in this patient, withdrawal symptoms were avoided following administration of a naloxone hydrochloride injection. This outcome likely reflects the benefit of administering naloxone hydrochloride to prevent withdrawal symptoms, as previously reported, followed by switching to fentanyl injections, which can be used relatively safely even in the presence of renal dysfunction.^1^

However, insurance coverage for serum cystatin C measurement is subject to certain restrictions. In Japan, insurance covers serum cystatin C measurement only once every 3 months and solely when renal dysfunction is suspected. Unlike serum creatinine, cystatin C levels cannot be measured frequently over a short period. Moreover, serum creatinine may not accurately reflect renal function in patients with sarcopenia, as demonstrated in our patient. Therefore, in undernourished patients with low BMI or those in poor general condition with low performance status, such as patients with terminal cancer, serum cystatin C should be measured at a single time point, either the day before initiating opioid therapy or upon hospitalization. Using this value as a reference, it is important to evaluate renal function comprehensively by monitoring relevant variables, including urine output, blood urea nitrogen, and serum creatinine.

The limitations of this study are as follows: First, this study was limited to a single patient. Respiratory depression is considered rare when morphine is used appropriately for the management of cancer pain.^2^ Therefore, this report is valuable as a detailed analysis of a patient who actually developed respiratory depression. Second, serum cystatin C could not be measured at the initiation of morphine hydrochloride injection, as no residual sample was available. Because of the missing data at this time point, it was difficult to determine whether renal function was already impaired at the initiation of morphine hydrochloride injections or if acute kidney injury developed during the administration period. However, based on the clinical course, renal injury may have developed sometime after hospitalization, as cancer pain remained relatively well controlled for 21 days following the switch to morphine hydrochloride injections. Third, blood levels of morphine and its active metabolites, M6G and M3G, could not be measured. Based on circumstantial evidence, it is speculated that M6G accumulation played a significant role, as respiratory depression was rapidly alleviated by naloxone hydrochloride, and morphine hydrochloride likely posed the greatest risk of respiratory depression among all drugs administered to this patient. It is highly likely that the diagnosis of sarcopenia via BIA combined with overestimation of renal function based on serum creatinine contributed to M6G accumulation, ultimately causing respiratory depression. A differential diagnosis for the cause of respiratory depression includes exacerbation of CO_2_ narcosis and the development or progression of brain metastases. However, neither condition was observed at the time of hospitalization, and invasive tests should be avoided in patients with terminal cancer whenever possible. Consequently, making a differential diagnosis based solely on the patient’s medical history and clinical course was challenging.

## Conclusion

Caution is warranted when assessing renal function in patients with terminal cancer using serum creatinine, as sarcopenia, a common complication, can lead to overestimation of renal function. In contrast, serum cystatin C–based assessment of renal function enables earlier and more accurate detection of renal impairment, suggesting its potential to guide the appropriate use of morphine.

## Supplementary Material

PDF-Japanese
