# Supplementary material for: Evaluation of renal function in a patient with terminal rectal cancer who developed morphine hydrochloride–induced respiratory depression
Source: Fujita Med J. 2026 May 14;12(3):227–30. doi: 10.20407/fmj.2025-021 (PMC13433044; doi:10.20407/fmj.2025-021)
Supplement: Supplementary file 1 — PDF-Japanese [file fmj-12-227_s1.pdf]

Fujita Medical Journal
Short Report

タイトル:

モルヒネ塩酸塩注射液により呼吸抑制を発現した終末期直腸がん患者 1 例の腎機能の検討

ランニングタイトル:

モルヒネにより呼吸抑制を発現した終末期がん患者 1 例の腎機能の検討

著者:

上葛義浩, PhD[1,2,*]
臼井正信, MD, PhD[2,*]
二村昭彦, PhD[2,3]
稲垣貴彦, BPharm[1]
[*]These two authors contributed equally to this article

所属機関:

[1] 藤田医科大学岡崎医療センター 薬剤部
[2] 藤田医科大学 医学部 外科・緩和医療学講座
[3] 鈴鹿医療科学大学 薬学部

責任著者:

上葛義浩
所属: 藤田医科大学岡崎医療センター 薬剤部／藤田医科大学 医学部 外科・緩和医療学講座
E-mail: yuekuzu@fujita-hu.ac.jp
住所: 〒444-0829 愛知県岡崎市針崎町西 2 丁目 6 番地 1
TEL: 0564-64-8205

臼井正信
所属: 藤田医科大学 医学部 外科・緩和医療学講座
E-mail: masanobu.usui@fujita-hu.ac.jp
住所: 〒470-1192 愛知県豊明市沓掛町田楽ケ窪 1 番地 98
TEL: 0562-93-2000

抄録

終末期がん患者は、悪液質の筋肉量減少により血清クレアチニンが低値となるため、血清クレアチニンによる腎機能評価では実際の値と乖離することが問題である。血清シスタチンCは、筋肉量減少時の腎機能評価に有用な指標だが、がん終末期に関する報告は少ない。今回、同一用量のモルヒネ塩酸塩注射液投与中に呼吸抑制を発現した終末期直腸がん患者1例の筋肉量を生体電気インピーダンス法で測定し、腎機能を詳細に検討したため報告する。

キーワード

終末期がん患者、サルコペニア、血清シスタチンC、eGFR、モルヒネ

本文

緒言

がん患者の薬物療法を適切に行うには、がんの病期、生命予後、全身状態、臓器機能の評価が重要である。これらを基に、最適な薬剤、投与方法を決定する。オピオイドは、がん患者の症状緩和に極めて有効だが、過量投与で発現する呼吸抑制は重篤で注意すべき副作用である[1)]。特に、モルヒネはがん疼痛だけでなく、呼吸困難の緩和に有用だが、腎機能低下時はモルヒネの活性代謝物であるモルヒネ-6-グルクロニド (morphine-6-glucuronide: M6G)、モルヒネ-3-グルクロニド (morphine-3-glucuronide: M3G) が蓄積するため、使用する際は減量などの慎重な対応が必要である[2,3)]。一般に、臨床現場における腎機能評価には、血清クレアチニンに基づく糸球体濾過量推算 (estimated glomerular filtration rate: eGFR) 式が推奨されている。しかし、血清クレアチニンは筋肉量の影響を受けやすいという限界がある。近年、終末期がん患者に発症する悪液質は、筋肉量の著明な減少を特徴とする病態であることが報告されている[4)]。従って、このような患者に対して血清クレアチニンに基づく eGFR 式をルーチンに用いることは、腎機能を過大評価する危険性がある。一方、血清シスタチンCは筋肉量の影響を受けにくいため、これに基づく eGFR 式は、筋肉量の減少した患者においてより適切な指標となり得る[5)]。以上より、終末期がん患者の腎機能を正確に評価するためには、筋肉量などの個別要因を考慮し、症例ごとに適切な eGFR 式を選択することが重要である。

本報告では、同一用量のモルヒネ塩酸塩注射液投与中に呼吸抑制を認めた終末期直腸がん患者1例において、生体電気インピーダンス (bioelectrical impedance analysis: BIA) 法により筋肉量を測定するとともに、腎機能を詳細に検討した結果について報告する。

方法

対象

患者: 70歳代、男性
疾患: 直腸がん
転移部位: 肝、肺、リンパ節
既往歴: 脳出血
現病歴: X-2年2月、直腸がんに対して、腹会陰式直腸切除術を施行した。同年6月、直腸がんの再発に伴い肝転移および仙骨全面リンパ節転移を認めたため、化学療法を開始した。X-1年1月には脳出血を発症し、化学療法を一時中断したが、同年12月に再開した。しかし、がんの進行および全身状態の悪化を認めたため、化学療法は中止さ

れた。X 年 1 月、緩和医療を目的として入院となった。

サルコペニアの評価: 身長 170 cm、体重 48.7 kg、body mass index (BMI) 16.9 kg/m^2、BIA 法 (InBody S10、株式会社インボディ・ジャパン) を用いて筋肉量を測定した結果、骨格筋量指数 (skeletal muscle mass index) は 5.4 kg/m^2 であり、カットオフ値 7.0 kg/m^2 未満であったことから、サルコペニアと診断した[6)]。

経過: 入院時、患者は絶食状態であり、静脈栄養によって管理されていた。脳出血の後遺症による両下肢の痺れのため、日中の大半を臥床して過ごしていた。進行がんであること、ならびに palliative performance scale が 20 であったことから、生命予後は約 1 ヶ月と予測され、がん終末期と診断された[7,8)]。腹部の内臓痛に対してフェンタニル貼付剤 (2mg/日) が使用されていたが、疼痛コントロール不十分であった。このため、入院当日にモルヒネ塩酸塩注射液 (20 mg/日) へオピオイドスイッチングを行った。その後、がん疼痛は numerical rating scale で 1 から 5 の間を推移し、比較的良好にコントロール可能であったため、同一用量のモルヒネ塩酸塩注射液で投与を継続した。第 21 病日、呼吸数の減少 (8 回/分未満)、昏睡、チアノーゼを認めた。モルヒネの過量による呼吸抑制が疑われ、生命への危険があると判断されたため、ナロキソン塩酸塩注射液を投与し、呼吸抑制は速やかに改善した[9)]。呼吸抑制発現時、膀胱留置カテーテルは挿入されておらず正確な尿量は確認できなかったが、浮腫、胸水、腹水などの体液貯留所見は認められなかった。なお、入院時から呼吸抑制発現時までに使用されていた薬剤は、モルヒネ塩酸塩注射液のほか、高カロリー輸液、脂肪乳剤、ファモチジン注射液、プロクロルペラジン注射液のみであった。呼吸抑制発現後は、フェンタニル注射液 (0.4 mg/日) へ再度オピオイドスイッチングを行い、以後、退薬症状は発現しなかった。第 34 病日、原疾患の進行により永眠された。

腎機能評価

モルヒネ塩酸塩注射液の投与開始時、開始後 (呼吸抑制発現前および発現時) における腎機能を、血清クレアチニン (酵素法で測定、測定機器: LABOSPECT 008 α (株式会社日立ハイテク)、試薬: L タイプワコー CRE・M (富士フイルム和光純薬株式会社)、基準値 0.65–1.07 mg/dL) および血清シスタチン C (呼吸抑制発現後に残余検体を用いラテックス免疫比濁法で測定、測定機器: LABOSPECT 008 α (株式会社日立ハイテク)、試薬: ノルディア シスタチン C (積水メディカル株式会社)、基準値 0.58–0.98 mg/L) を用いて評価した。これらのデータに基づき、それぞれの項目から推算される eGFR を算出し、呼吸抑制の発現との関係を経時的に検討した。eGFR の算出には、次の 4 つの推算式を用いた。

血清クレアチニンによる日本人の eGFR 式 ($eGFR_{Scr}$)[10)]

$$\mathrm{eGFR_{Scr}}\ (\mathrm{mL/min/1.73m^2}) = 194 \times 血清クレアチニン\ (\mathrm{mg/dL})^{-1.094} \times 年齢\ (歳)^{-0.287} \times 0.739\ (女性の場合)$$

血清シスタチン C による日本人の eGFR 式 ($eGFR_{Scys}$)[11)]

$$\mathrm{eGFR_{Scys}}\ (\mathrm{mL/min/1.73m^2})$$

$$= \left\{104 \times \text{血清シスタチン C}\ (\mathrm{mg/L})^{-1.019} \times 0.996^{\text{年齢 (歳)}} \times 0.929\ (\text{女性の場合})\right\} - 8$$

血清クレアチニンと血清シスタチン C による日本人の複合式 ($\mathrm{eGFR_{Scr,Scys}}$)[11)]

$$\mathrm{eGFR_{Scr,Scys}}\ (\mathrm{mL/min/1.73m^2}) = \frac{\mathrm{eGFR_{Scr}}\ (\mathrm{mL/min/1.73m^2}) + \mathrm{eGFR_{Scys}}\ (\mathrm{mL/min/1.73m^2})}{2}$$

Cockcroft-Gault 式の eGFR 補正値 ($\mathrm{eGFR_{CG}}$)[10,12,13)]

$$\mathrm{eGFR_{CG}}\ (\mathrm{mL/min/1.73m^2})$$

$$= \frac{\{140 - \text{年齢}\ (\text{歳})\} \times \text{体重}\ (\mathrm{kg})}{72 \times \text{血清クレアチニン}\ (\mathrm{mg/dL})} \times \frac{1.73}{\text{体表面積}}\ (\mathrm{m^2}) \times 0.789\ (\text{補正係数}) \times 0.85\ (\text{女性の場合})$$

倫理的配慮

本研究は、藤田医科大学の倫理審査委員会によって承認されたプロトコルに従って実施した。

結果

本研究における血清シスタチン C の測定は、呼吸抑制発現後に実施した。しかし、モルヒネ塩酸塩注射液の投与開始時は、残余検体がなかったため測定することができなかった。図 1 に血清クレアチニン、血清シスタチン C の測定値を示す。モルヒネ塩酸塩注射液の投与開始時、呼吸抑制発現前、発現時の血清クレアチニン (mg/dL) は 0.52、0.72、1.82 であり、血清シスタチン C (mg/L) は測定不可、2.08、2.55 であった。図 2 に eGFR 式の推算値 ($\mathrm{mL/min/1.73m^2}$) を示す。モルヒネ塩酸塩注射液の投与開始時、呼吸抑制発現前、発現時の血清クレアチニンによる日本人の eGFR 式 ($\mathrm{eGFR_{Scr}}$) を用いた場合は 116.3、81.4、29.5 であり、血清シスタチン C による日本人の eGFR 式 ($\mathrm{eGFR_{Scys}}$) を用いた場合は、血清シスタチン C 測定不可、29.0、22.0 であった。$\mathrm{eGFR_{Scys}}$ は呼吸抑制発現の少なくとも 3 日前から腎機能低下を示唆していたが、$\mathrm{eGFR_{Scr}}$ は腎機能を過大評価していた。血清クレアチニンと血清シスタチン C による日本人の複合式 ($\mathrm{eGFR_{Scr,Scys}}$) を用いた場合は、血清シスタチン C 測定不可、55.2、25.8、Cockcroft-Gault 式の eGFR 補正値 ($\mathrm{eGFR_{CG}}$) を用いた場合は 77.8、56.2、22.2 であり、eGFR 式、Cockcroft-Gault 式に血清クレアチニンを含むことで腎機能を過大評価する傾向であった。

考察

本研究は、同一用量のモルヒネ塩酸塩注射液投与中に呼吸抑制を発現した終末期直腸がん患者 1 例を対象に BIA 法により筋肉量を測定し、腎機能を詳細に検討した報告である。その結果、血清シスタチン C による腎機能評価が、終末期がん患者に対する

モルヒネの適正使用に有用である可能性が示唆された。

本研究における最も重要な知見は、サルコペニアを合併した終末期がん患者においては、推算式に血清クレアチニンを含む eGFR 式、Cockcroft-Gault 式を用いると腎機能を過大評価される点である。一般の臨床現場では、血液検査値からは血清クレアチニン単独、あるいは、血清クレアチニンと血清シスタチン C の両方の値を用いることで腎機能を正確に推算することが可能である [10,11]。しかしながら、終末期がん患者の場合は、悪液質に伴う筋肉量の著明な減少がみられることから、推算式に血清クレアチニンを含まない $eGFR_{Scys}$ が正確な腎機能評価に有用であると考えられる。

次に注目すべき点は、$eGFR_{Scys}$ は 29.0 mL/min/1.73m^2 と呼吸抑制発現 3 日前から腎機能低下を示していたのに対し、$eGFR_{Scr}$ は 81.4 mL/min/1.73m^2 と腎機能を過大評価していたことである。この eGFR の乖離値は 52.4 mL/min/1.73m^2 であり、オピオイド選択および投与量に関する臨床判断を行う上で大きな影響を及ぼすと考えられる。ガイドラインでは、腎機能障害患者に対して、モルヒネはできるだけ用いないことが望ましいとされること、使用する際も減量、あるいは、投与間隔を延長することが示されている [2]。本症例では、同一用量のモルヒネ塩酸塩注射液投与中に呼吸抑制を発現したが、血清シスタチン C により腎機能低下を早期に把握できていれば、モルヒネの減量や他のオピオイドへの変更などの判断が可能であった可能性が考えられる。さらに、臨死期は、モルヒネのクリアランスが低下することが報告されており、腎機能低下の兆候を早期に把握することは、薬物療法上、極めて重要である [14]。

また、本症例では、ナロキソン塩酸塩注射液投与後に退薬症状を回避できた点も注目される。これは、われわれの過去の報告を参考に、ナロキソン塩酸塩注射液投与後、腎機能低下時でも比較的安全に使用可能なフェンタニル注射液を使用して、慎重にオピオイドスイッチングを実施したことが離脱症状の回避に有用であったと考えられる [1]。

一方で、血清シスタチン C の測定には、保険上の制限がある点には留意すべきである。本邦では保険診療上、腎機能低下が疑われる場合に限り、3 ヶ月に 1 回のみ測定が認められている。血清シスタチン C は、血清クレアチニンのように短期間に複数回測定することが困難であり、血清クレアチニンでは、本症例のようなサルコペニア患者の場合は、実際の腎機能を反映しない可能性がある。従って、がん終末期のような BMI が低い低栄養患者、performance status が低い全身状態不良患者の腎機能評価を行う場合は、オピオイド導入前日、あるいは、入院時に 1 ポイントの血清シスタチン C を測定する。そして、その値を基準として、尿量、血中尿素窒素、血清クレアチニンの変動などから、腎機能を総合的に評価することが重要と考えられる。

本研究の限界は、以下のことがあげられる。第 1 は、本研究の対象が 1 例ということである。一般にがん疼痛治療を目的としてモルヒネを適切に使用する限り、呼吸抑制を発現することは稀とされている [2]。そのため、実際に呼吸抑制を発現した症例を詳細に解析し報告したことには意義がある。第 2 は、モルヒネ塩酸塩注射液投与開始時の血清シスタチン C が、残余検体がなかったため測定不可能であったことである。このデータ欠損により、モルヒネ塩酸塩注射液投与開始時点ですでに腎機能低下が存在していたのか、あるいは、投与期間中に急性腎障害が進行したのかの判断が困難となっている。しかし、臨床経過からの推測にはなるが、入院後にモルヒネ塩酸塩注射液へオピオイドスイッチングを行い、21 日間はがん疼痛が比較的良好にコントロール可能であったことから、入院後しばらく経過してから腎障害が発現した可能性が示唆される。第 3 は、モルヒネおよびその活性代謝物である M6G、M3G の血中濃度測定が実施できな

かったことである。状況証拠に基づく推論ではあるが、ナロキソン塩酸塩注射液の使用により呼吸抑制が改善したこと、本症例の使用薬剤ではモルヒネ塩酸塩注射液が呼吸抑制の原因として最も危険性が高いと考えられたことから、M6G の蓄積が強く示唆される。さらに、BIA 法によりサルコペニアと診断したこと、血清クレアチニンにより腎機能を過大評価した結果より、M6G が蓄積し、呼吸抑制に至った可能性が極めて高いと考えられる。また、他に呼吸抑制をきたしうる鑑別疾患として CO_2 ナルコーシスの増悪、脳転移の出現および増悪の可能性があげられる。しかし、当院入院時においてはどちらも認められなかったこと、がん終末期であり侵襲を伴う検査は可能な限り避けるのが望ましい状況であることから、現病歴や臨床経過のみでこれらを除外鑑別するのは難しいと考えられる。

結論

終末期がん患者は、サルコペニアを合併していることが多く、血清クレアチニンを使用した腎機能評価は過大評価の可能性があるため注意が必要である。一方、血清シスタチン C による腎機能評価は、腎機能低下をより早期かつ正確に評価することが可能であり、モルヒネの適正使用につながる可能性が示唆される。

利益相反

すべての著者において、開示すべき利益相反はない。

引用文献

1) Uekuzu Y, Higashiguchi T, Futamura A, Ito A, Mori N, Murai M, Ohara H, Awa H, Chihara T. A Clinical Study on Administration of Opioid Antagonists in Terminal Cancer Patients: 7 Patients Receiving Opioid Antagonists Following Opioids among 2443 Terminal Cancer Patients Receiving Opioids. Biol Pharm Bull 2017; 40: 278–83.
2) Japanese Society for Palliative Medicine. Clinical guidelines for cancer pain management. 3rd Edition. Tokyo: KANEHARA & Co., Ltd.; 2020: 53–66 (in Japanese).
3) Japanese Society for Palliative Medicine. Clinical Guidelines for the Treatment of Dyspnea in Advanced Diseases. 2023 ed. Tokyo: KANEHARA & Co., Ltd.; 2023: 131–5 (in Japanese).
4) Fearon K, Strasser F, Anker SD, et al. Definition and classification of cancer cachexia: an international consensus. Lancet Oncol 2011: 12; 489–95.
5) Uekuzu Y, Higashiguchi T, Futamura A, Chihara T, Usui M. Influence of muscle mass on the estimation of glomerular filtration rate in Japanese terminal cancer patients. Clin Exp Nephrol 2020; 24: 876–84.
6) Chen LK, Woo J, Assantachai P, et al. Asian Working Group for Sarcopenia: 2019 Consensus Update on Sarcopenia Diagnosis and Treatment. J Am Med Dir Assoc 2020; 21: 300–7.
7) Anderson F, Downing GM, Hill J, Casorso L, Lerch N. Palliative performance scale (PPS): a new tool. J Palliat Care 1996; 12: 5–11.
8) Higashiguchi T, Ikegaki J, Sobue K, Tamura Y, Nakajima N, Futamura A, Miyashita M, Mori N, Inui A, Ohta K, Hosokawa T. Guidelines for parenteral fluid management for terminal cancer patients. Jpn J Clin Oncol 2016; 46: 986–92.
9) Sweeney C, Bogan C. Textbook of Palliative Medicine. London: Hodder Arnold; 2006: 390–401.
10) Matsuo S, Imai E, Horio M, Yasuda Y, Tomita K, Nitta K, Yamagata K, Tomino Y, Yokoyama H, Hishida A. Revised equations for estimated GFR from serum creatinine in Japan. Am J Kidney Dis 2009; 53: 982–92.

11) Horio M, Imai E, Yasuda Y, Watanabe T, Matsuo S. GFR estimation using standardized serum cystatin C in Japan. Am J Kidney Dis 2013; 61: 197–203.
12) Cockcroft DW, Gault MH. Prediction of creatinine clearance from serum creatinine. Nephron 1976; 16: 31–41.
13) Du Bois D, Du Bois EF. A formula to estimate the approximate surface area if height and weight be known. 1916. Nutrition 1989; 5: 303–11; discussion 312–3.
14) Franken LG, Masman AD, de Winter BC, Koch BC, Baar FP, Tibboel D, van Gelder T, Mathot RA. Pharmacokinetics of Morphine, Morphine-3-Glucuronide and Morphine-6-Glucuronide in Terminally Ill Adult Patients. Clin Pharmacokinet 2016; 55: 697–709.

図のキャプション

図 1. 腎機能 (血清クレアチニン、血清シスタチン C) と呼吸抑制発現の経時的変化
On admission: At the start of morphine administration
Day 18: Three days before onset of respiratory depression
Day 21: When respiratory depression occurs

図 2. 腎機能 (eGFR) と呼吸抑制発現の経時的変化
On admission: At the start of morphine administration
Day 18: Three days before onset of respiratory depression
Day 21: When respiratory depression occurs
eGFR: 推算糸球体濾過量 (mL/min/1.73 m^2)
$eGFR_{Scr}$: 血清クレアチニンによる日本人の eGFR 式
$eGFR_{CG}$: Cockcroft-Gault 式の eGFR 補正値
$eGFR_{Scr,Scys}$: 血清クレアチニンと血清シスタチン C による日本人の複合式
$eGFR_{Scys}$: 血清シスタチン C による日本人の eGFR 式

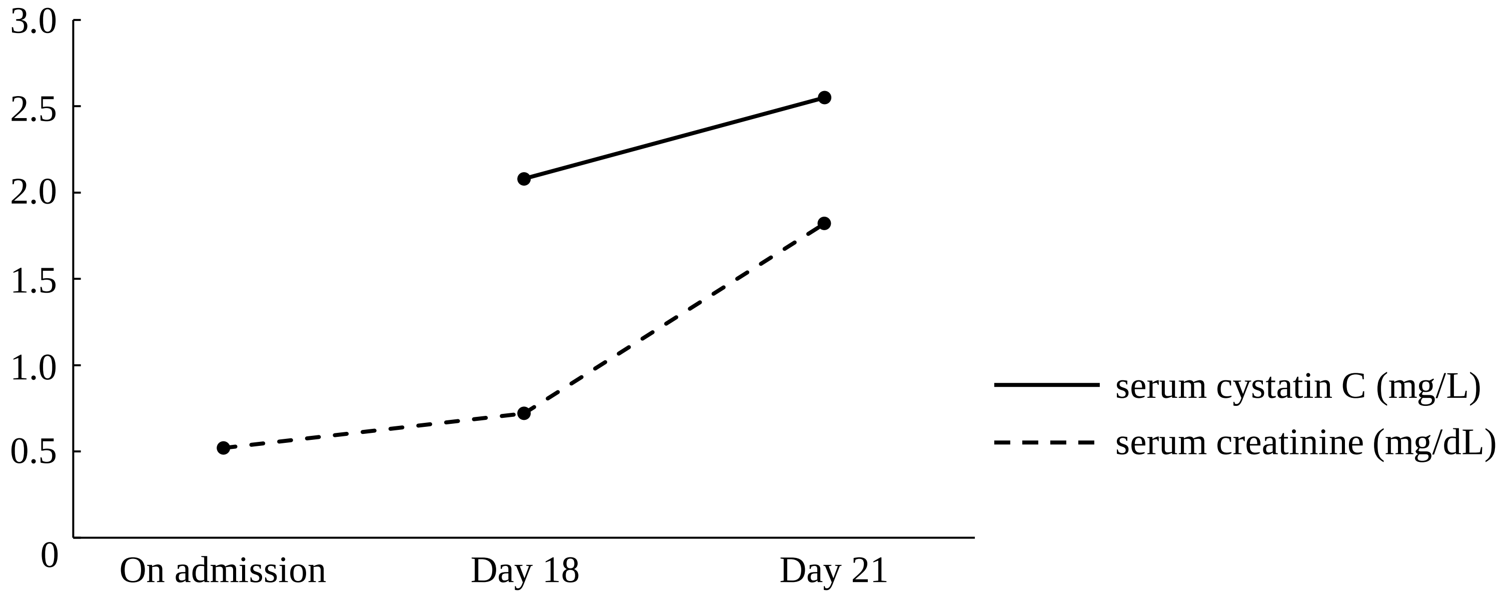
3.0
2.5
2.0
1.5
1.0
0.5
0
On admission
Day 18
Day 21
serum cystatin C (mg/L)
serum creatinine (mg/dL)

Fig. 1

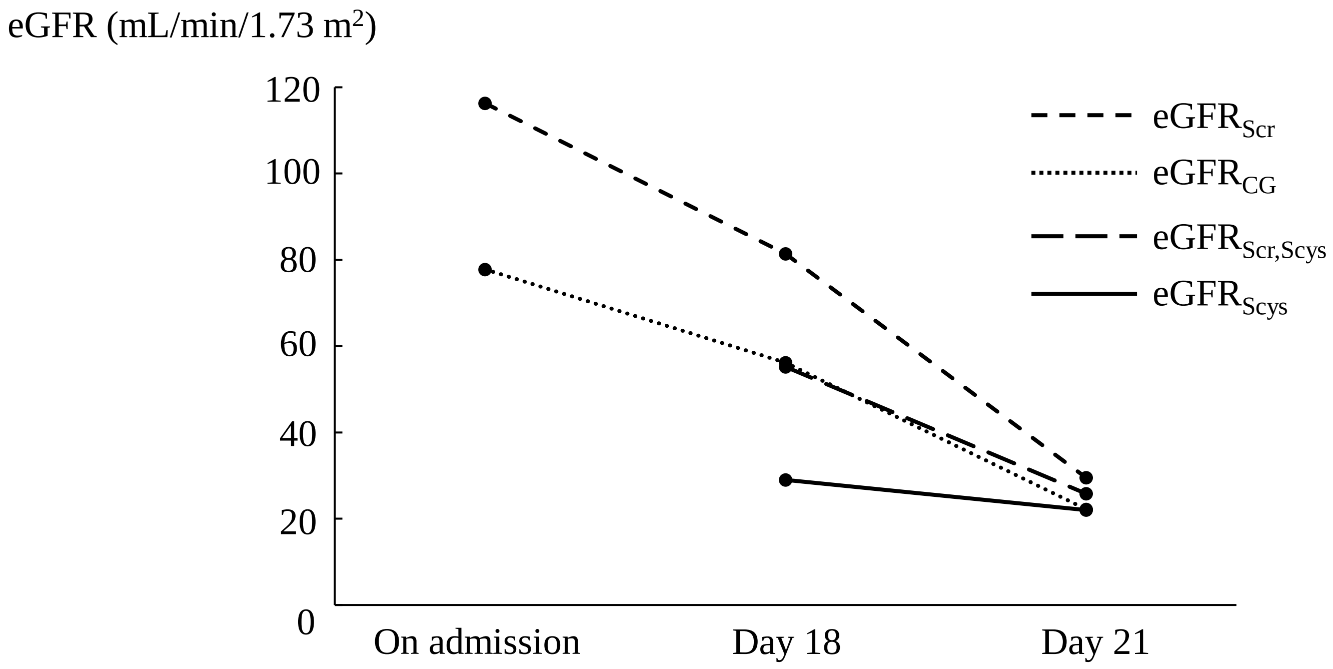
eGFR (mL/min/1.73 m²)
120
100
80
60
40
20
0
On admission
Day 18
Day 21
eGFR$_{Scr}$
eGFR$_{CG}$
eGFR$_{Scr,Scys}$
eGFR$_{Scys}$

Fig. 2
